# Covalent Organic Frameworks: A Promising Materials Platform for Photocatalytic CO_2_ Reductions

**DOI:** 10.3390/molecules25102425

**Published:** 2020-05-22

**Authors:** Jundan Li, Dongni Zhao, Jiangqun Liu, Anan Liu, Dongge Ma

**Affiliations:** 1 School of Science, Beijing Technology and Business University, Beijing 100048, China; 1930102009@st.btbu.edu.cn (J.L.); celinazhao07@gmail.com (D.Z.); liujiangqunBTBU@163.com (J.L.); 2 Basic Experimental Center for Natural Science, University of Science and Technology Beijing, Beijing 100083, China; liuanan@ustb.edu.cn

**Keywords:** covalent organic frameworks, CO_2_ reduction, photocatalysis, functional materials design

## Abstract

Covalent organic frameworks (COFs) are a kind of porous crystalline polymeric material. They are constructed by organic module units connected with strong covalent bonds extending in two or three dimensions. COFs possess the advantages of low-density, large specific surface area, high thermal stability, developed pore-structure, long-range order, good crystallinity, and the excellent tunability of the monomer units and the linking reticular chemistry. These features endowed COFs with the ability to be applied in a plethora of applications, ranging from adsorption and separation, sensing, catalysis, optoelectronics, energy storage, mass transport, etc. In this paper, we will review the recent progress of COFs materials applied in photocatalytic CO_2_ reduction. The state-of-the-art paragon examples and the current challenges will be discussed in detail. The future direction in this research field will be finally outlooked.

## 1. Introduction

In recent years, the anthropogenic consumption of fossil fuels increased with an exponential growth rate. This led to energy exhaustion, environmental pollution and the warming-up effect by CO_2_ emission. All of these have severely threatened the sustainable and harmonious development of human society. The environment and energy crises have become the most lethal enemies for human-beings. Developing efficient approaches to reduce the enormous amount of carbon dioxide being discharged into the environment has become an urgent need. In nature, plants use sunlight to convert carbon dioxide into glucose by photosynthesis [1]. Inspired by this, chemists began to focus on the effective methods to use solar energy to convert CO_2_ into valuable chemicals about a century ago [2,3]. Due to the more and more serious situations facing to the human society, converting CO_2_ into fuels, other useful stock and fine chemicals has become one of the best options and been considered as “the holy grail” to reduce green-house gas emission, counter the energy shortage and ameliorate the environment pollution. It can be seen as the solution to “kill three birds with one stone” [4,5,6,7].

CO_2_ reduction reaction is a considerably endothermic process, which makes it unfavorable in thermodynamics. Compared with water-splitting reaction, the CO_2_ reduction reaction requires more in-put energy (see Equations (1)–(6) below). Different from common thermocatalytic CO_2_ reduction, photocatalytic materials not only need to convert solar energy into electron/hole pair possessing oxidizing and reducing ability, but also should be capable of reducing the activation energy for CO_2_ reduction. Photocatalytic CO_2_ reduction is a multi-electron transfer process which produces different reduction products at varied redox potentials as shown in Table 1 below. The key to obtain a certain reduction product is to choose the photocatalytic materials with matched energy band position and E_g_. However, this is considered to be the most challenging point in CO_2_ reduction. The following processes shown in Table 1 often occur simultaneously, leading to the formation of multiple reduction products. Moreover, the proton can also act as the electron-acceptor to generate H_2_ by-product, worsening the CO_2_ reduction selectivity.

H_2_O(l) → H_2_(g) + 1/2O_2_(g), ΔG^0^ = 237 kJ/mol, ΔE^0^ = 1.23 V. (1)

CO_2_(g) → CO(g) + 1/2O_2_(g), ΔG^0^ = 257 kJ/mol, ΔE^0^ = 1.33 V. (2)

CO_2_(g) + H_2_O(l) → HCOOH(l) + 1/2O_2_(g), ΔG^0^ = 286 kJ/mol, ΔE^0^ = 1.48 V. (3)

CO_2_(g) + H_2_O(l) → HCHO(l) + O_2_(g), ΔG^0^ = 522 kJ/mol, ΔE^0^ = 1.35 V. (4)

CO_2_(g) + 2H_2_O(l) → CH_3_OH(l) + 3/2O_2_(g), ΔG^0^ = 703 kJ/mol, ΔE^0^ = 1.21 V. (5)

CO_2_(g) + 2H_2_O(l) → CH_4_(g) + 2O_2_(g), ΔG^0^ = 818 kJ/mol, ΔE^0^ = 1.06 V. (6)

In order to effectively utilize solar energy to convert CO_2_ to useful chemicals, a key issue should be addressed first; that is, to design and prepare highly-efficient “artificial leaf” materials which act as the antenna to absorb solar energy, transform the solar energy to chemical energy and drive the kinetically sluggish and thermodynamic unfavored CO_2_ reduction reaction. Since the first report by Inoue and co-workers in 1979 of successfully transforming CO_2_ into various hydrocarbons in aqueous solution using semiconductors [8] under sunlight illumination, a renaissance of photocatalytic CO_2_ reduction surged. Various inorganic semiconductors were extensively investigated to screen efficient CO_2_ photocatalytic reduction catalysts [9,10,11,12,13]. Besides photocatalysts themselves, the choice of electron-donor, photosensitizer and sacrificial reagents can also largely affect the overall efficiency of CO_2_ photocatalytic reduction. Using TEOA, ascorbic acid or alcohol as electron-donor and sacrificial reagent would usually provide a much higher CO_2_ reduction yield than H_2_O because of their lower oxidizing potential and rapid electron-transfer kinetics, but exploiting H_2_O as electron-donor and avoiding sacrificial reagent is more ideal from the consideration of sustainability. Although applying photosensitizer such as Ru(bpy)_3_Cl_2_ can remarkably increase CO_2_ photocatalytic reduction efficiency by more effective light-harvesting, combining the photocatalysis and photosensitization function in semiconductor photocatalyst itself without the requirement of additional photosensitizer would be preferable due to the avoidance of the use of expensive and toxic transition-metal complex. If more delicate design and structural optimization of photocatalysts materials can be achieved, the ultimate goal of applying H_2_O as the only electron-donor without extra sacrificial reagent and using semiconductor as the sole photocatalyst without noble-metal photosensitizer is not difficult to realize.

Up to now, homogeneous transition-metal complex based catalysts [14,15], heterogeneous inorganic semiconductor catalysts [16,17], and recently developed inorganic-organic hybrid metal-organic frameworks (MOFs) materials [18,19,20] have found applications in CO_2_ photocatalytic transformations. MOFs materials combined the long-comings of homogeneous organometallic-based molecular catalysts and inorganic semiconductor photocatalysts. On one hand, the metal centre and organic ligand can facilitate efficient light harvesting. On the other hand, the transition metal plays a pivotal role in the activation and transformation of CO_2_ due to its high affinity towards CO_2_ through metal-carbonyl coordination interaction. Moreover, the metal centre can experience fruitful oxidative addition and reductive elimination steps, and stabilize the intermediate state during CO_2_ reduction. Further, MOFs are highly porous materials with a huge amount of CO_2_ docking sites. The well-defined structure with high crystallinity confirmed the establishment of accurate structure-activity relationships (SARS) as crystalline inorganic semiconductor photocatalysts do. However, since most MOFs materials are composed by weak coordination bonds, only few are stable enough to endure the aqueous condition and achieve the ideal goal of reducing CO_2_ with H_2_O as the electron donor. Since, in 2005, Yaghi and co-workers first synthesized COF-1 material by the self-condensing of phenyl diboronic acid forming boroxine bonds into a 2D porous polymer with a long-range ordered structure, COFs materials have seen a burgeoning development [21]. Compared with their MOFs counterparts, COFs materials, which are connected via strong covalent bonds, are more difficult to obtain a good macroscopic crystal [22]. By thermodynamically controlled reversible chemistry based on “error-checking” and “proof-reading” mechanisms, a number of COFs materials were synthesized by various methods such as solvothermal, ultrasonic treatment, ionothermal, mechanochemcal treatment, room-temperature Lewis-acid catalysis conditions and the interfacial synthesis for COFs films. In comparison with other organic semiconductor-based photocatalyst materials such as carbon nitride, COFs materials possess the tunable skeleton and pore structures to offer plenty of docking sites for catalytically active species and provide a large surface area to fix carbon dioxide [23]. Compared with other porous polymeric materials such as hyper-crosslinked polymer, conjugated microporous polymer, COFs materials own long-range ordered crystalline structure and its structures are clearer than other amorphous porous polymeric materials, which facilitates the demonstration of structure-activity-relationship (SARS) and further optimizing of its performance. Moreover, the pore engineering provides specific interaction with CO_2_ by non-covalent bonding, which facilitates its adsorption and reaction in the nano-channels. The pore structure can act as a nano-reactor to shape a confined micro-environment to facilitate the CO_2_ adsorption and reduction products desorption. This selective mass transport flow will highly benefit the catalyst performance. Besides, COFs ordered π-array structure can provide a pre-organized channel for rapid and efficient charge-carrier transport [24], which will further improve the activity of the photocatalytic reduction of CO_2_. By condensing electron-rich and electron-deficient moiety into a bicontinious alternative structure, the 1D column array of donor-donor and acceptor-acceptor can be thus formed [25]. This super-heterojunction structure possesses better charge-transfer and collection behavior in comparison with inorganic semiconductors and common organic 1D polymeric material. Besides these two advantages, the tunability of COFs structure can accommodate different π-conjugated module, electron donor and acceptor groups, which will form excellent antennae and achieve the effective harvesting of the whole sunlight spectrum extending from ultra-violet (UV), visible-light (Vis) to near-infrared light (NIR). These crucial factors, the superb charge carrier transport and collection, the light harvesting, and the adsorptive performance ensure COFs to be promising material platforms for photocatalytic CO_2_ reduction [26].

There have been several excellent reviews on COFs materials application in photocatalysis [27], mainly focused on photocatalytic hydrogen evolution [28] or organic transformations [29]. This review will focus on the applications of COFs-based photocatalysts for the CO_2_ reduction field. The discussion will be divided into three parts according to the photocatalyst system. Firstly, we will focus on metalated COF photocatalysts and hybrid photocatalysts of COFs materials with homogeneous molecular CO_2_ reduction catalyst. The second part will discuss pure COFs photocatalysts for CO_2_ reduction without transition-metal moiety. Besides, the covalent triazine frameworks (CTFs) which are capable of photocatalytic CO_2_ reduction will also be incorporated. Finally, the current challenge and the future direction of this field will be presented in the conclusion.

## 2. COFs Application for Photocatalytic CO_2_ Reduction

### 2.1. Metalated COFs and Hybrid COF Photocatalyst Systems with Homogeneous CO_2_ Reduction Catalysts

Compared with pure COFs photocatalytic CO_2_ reduction materials, metalated COFs and hybrid COF catalyst systems conjugated with homogeneous metal-complex molecular CO_2_ reduction catalysts often exhibited better activity. This activity enhancement was mainly attributed to the presence of transition-metal elements, which usually possess outstanding CO_2_ adsorption and catalytic capability or have the ability to considerably strengthen the photo-induced charge generation and accelerate charge-transfer process.

**Figure 1:**
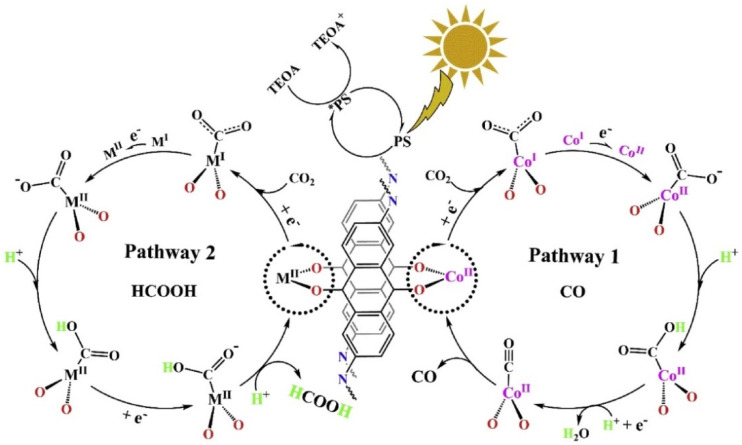
Proposed mechanism for the photocatalytic reduction of CO_2_ with DQTP-COF-M ((M = Zn, Ni and Co). Copied with permission from Elsevier 2019 [30].

In 2019, Lan et al., developed the first metalated COF material for heterogeneous photocatalytic CO_2_ reduction. The authors reticulated 2,6-diaminoanthraquinone (DQ) with 2,4,6-triformylphloroglucinol (TP) in DMA (*N*,*N*-dimethylacetamide)/1,3,5-trimethylbenze/6M acetic acid mixed solvent heated at 120 °C for 72 h [30]. The schematic diagram of the reaction mechanism is demonstrated in Figure 1. The as-prepared 2D-anthraquinone-based DQTP-COF possessed plentiful metal coordinating sites via anthraquinone O atoms. Upon the sonication and heating in the aqueous solution with cobalt salt, Co(II) ion was incorporated into the COF structure. The as-prepared DQTP-COF-Co material showed good CO_2_ reduction ability under visible-light irradiation (λ ≥ 420 nm) sensitized by Ru(bpy)_3_Cl_2_ with triethanolamine (TEOA) as electron donor. The formation rate of CO was as high as 1.02 × 10^3^ μmol g^−1^ h^−1^, which was much higher than the non-metalated DQTP-COF, DQTP-Ni-COF and DQTP-Zn-COF samples. The selectivity for CO over H_2_ was moderate (59.4%). However, when Zn(II) ion was loaded into the DQTP-COF structure, HCOOH was generated with an excellent selectivity up to 90% and a formation rate of 152.5 μmol g^−1^ h^−1^. The authors proposed a “two-pathway” mechanism for this distinguishing selectivity. Two pathways forming either HCOOH or CO share the same intermediate, i.e., M-COOH (see Figure 1). If the coordination environment for -COOH was electron-rich, the C-O bond was prone to be cleaved forming CO product, while the electron-deficiency environment would facilitate the HCOOH formation via proton-coupled-electron-transfer process (PCET), which by-passed the C-O cleavage pathway. Since Co is a good π-electron donor, CO was the main product; while for Zn-based COF, the π-electron acceptor metal centre would promote the formation of HCOOH. While for Ni-COF, which was neither electron-rich nor deficient, the generation rate of CO and HCOOH was almost identical. Lan’s report was the first example using metalated-COF material as heterogeneous CO_2_ photocatalyst.

**Figure 2:**
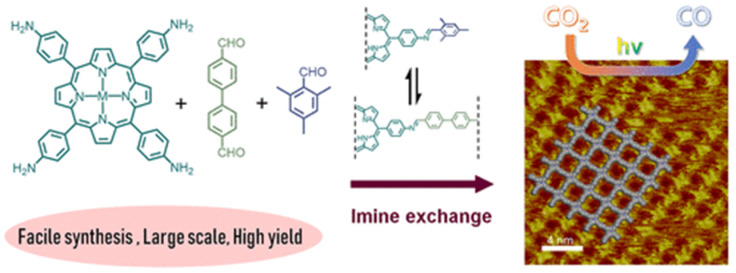
Schematic illustration of synthesis of the COF-367 NSs for CO_2_ photoreduction to CO and its STM image. Copied with permission from ACS 2020 [31].

Recently, Jiang et al., discovered that via a bottom-up preparation approach, COF-367-Co nanosheet (NS) exhibited considerably high activity for photocatalytic CO_2_ reduction to CO under visible-light irradiation conditions [31], and the preparation procedures are shown in Figure 2. The authors discovered that during the condensation of 5,10,15,20-tetrakis(4-aminophenyl)porphinato]cobalt (Co-TAP) and biphenyl-4,4′-dicarboxaldehyde (BPDA) to fabricate COF-367-Co, the addition of excess amount of bulky BPDA analogue 2,4,6-trimethylbenzaldehyde (TBA) would induce the anisotropical growth of COF crystallite. The bulky additive can inhibit growth in the perpendicular direction of the nanocrystallite. Thus, the ultra-thin COF-367-Co nanosheet (NS) could be prepared even in gram scale with more exposed catalytic active sites. The COF-367-Co NS exhibited superior photocatalytic performance in comparison with the bulk COF-367-Co powder with 10,672 μmol g^−1^ h^−1^ CO yield and a selectivity of ca. 78% in aqueous solution sensitized by Ru(bpy)_3_Cl_2_ under λ ≥ 420 nm irradiation with ascorbic acid as electron donor. When bulk COF-367-Co powder material was used under other identical conditions, in sharp contrast, only 65 μmol g^−1^ h^−1^ of CO and 514 μmol g^−1^ h^−1^ of H_2_ were evolved. This great improvement of activity was attributed to the ultra-thin 2D NS structure which facilitated the photo-induced electron transfer evidenced by the transient photoluminescence spectrometry and ultra-fast transient absorption experiments results. Further, the DFT theoretical calculation results demonstrated that cobalt atom plays the role as both CO_2_ adsorption and activation sites, generates the key intermediate COOH* with a 0.47 eV low energy barrier, while a much higher 0.74 eV energy barrier is required for proton reduction. This sharp difference accounted for the good selectivity of CO_2_ reduction over proton reduction. This example still remains as the record-high activity for photocatalytic CO_2_ reduction up till now in spite of the use of noble-metal sensitizer Ru(bpy)_3_Cl_2_ and ascorbic acid electron-donor.

**Figure 3:**
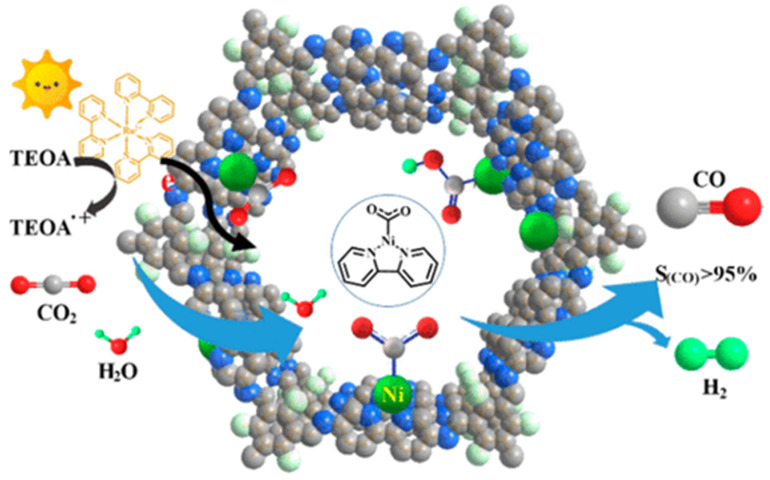
Schematic diagram of photocatalytic selective reduction of CO_2_ over Ni-TpBpy. Copied with permission from ACS 2019 [32].

Almost at the same time with Lan’s report on DQTP-COF-Co and DQTP-COF-Zn CO_2_ photoreduction catalysts, Zou et al., discovered that nickel single site catalyst could be loaded on TpBp-COF supporter to construct metalated COF photocatalyst for efficient CO_2_ reduction [32], as demonstrated in Figure 3. The authors demonstrated that the condensation of TP and 5,5′-diamino-2,2′-bipyridine (BP) under solvothermal conditions generated TpBp-COF material. Moreover, the Ni single site catalyst could be incorporated into the COF structure only through simple impregnation treatment of TpBp-COF with Ni(ClO_4_)_2_. The state of single Ni sites on COF supporter was characterized by various techniques including aberration-corrected high-angle annular dark-field scanning transmission electron microscopy (HAADF-STEM). The as-synthesized TpBp-COF-Ni single site material exhibited considerably good performance for photocatalytic CO_2_ reduction under visible-light irradiation sensitized by Ru(bpy)_3_Cl_2_ with TEOA as electron donor. The CO formation rate was as high as 966 μmol g^−1^ h^−1^. An almost perfect 96% CO over H_2_ and other reduction products selectivity was achieved. Although TpBp-COF-Co provided higher reduction activity compared with TpBp-COF-Ni, the CO selectivity was poorer in comparison with Ni-based catalyst. The authors applied DFT calculation to investigate the origin of this extraordinary activity and selectivity. The calculation results indicated that the addition of H^+^ to L_2_Ni (L means ligand) could not occur easily due to the large 141.4 kcal mol^−1^ energy barrier, while the formation of L_2_Ni-CO_2_ adduct only required activation energy of 3.2 kcal mol^−1^. The following steps—including the sequential proton and electron addition to L_2_Ni-CO_2_, the transformation of Ni-COOH to Ni-CO, and the release of CO—only needed to overcome considerably smaller energy barriers. Moreover, since the ΔG of COF-Ni adduct formation was less than Ni(bpy)_2_, the kinetics of CO_2_ reduction at the Ni single site supported on TpBp-COF were more favorable than H^+^ reduction. Zou’s example realized the first single metal site catalyst supported on COF to fulfill photocatalytic CO_2_ reduction with high reduction efficiency and perfect product selectivity.

**Figure 4:**
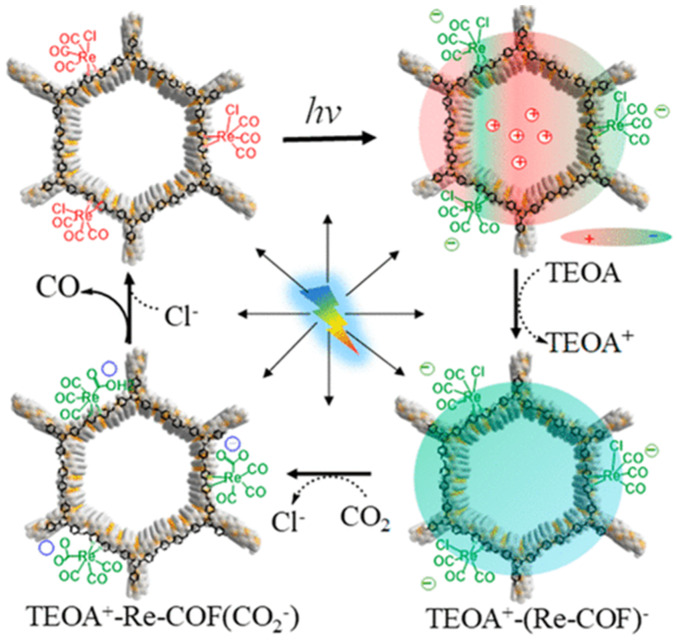
Proposed catalytic mechanism of Re-COF. Copied with permission from ACS 2019 [33].

Besides metalated-COF photocatalytic CO_2_ reduction materials, the conjunction of COFs materials with other homogeneous CO_2_ reduction catalysts is another strategy to achieve highly efficient and selective CO_2_ reduction under visible-light irradiation. Noble-metal Re-complex molecular catalyst showed outstanding efficiency for CO_2_ photocatalytic reduction when combined with COF materials. In 2018, Huang et al. reported the first rhenium-COF-based hybrid photocatalyst system, which exhibited superior selectivity up to 98% towards CO_2_ reduction to CO and high activity, with 15 mmol g^−1^ CO steady generation for more than 20 h, which accounted for a TON 48 and 22 times better than its homogeneous counterpart [33]. Their proposed catalysis mechanism is shown in Figure 4. This rhenium-COF hybrid photocatalytic system was constructed via the postsynthetic modification of the BPDA-TTA-COF which was generated by the Schiff base condensation reaction of 2,2-bipyridyl-5,5-dialdehyde (BPDA) and 4,4′,4″-(1,3,5-triazine-2,4,6-triyl) trianiline (TTA) under solvothermal condition. Re-moiety was introduced by the ligand exchange procedure of bipyridine ligand in COF with chloride in the Re precursor Re(CO)_5_Cl. The as-synthesized Re-COF was characterized by various spectrometric methods including FT-IR, XRD, BET and UV-Vis. To demonstrate the photophysical and photochemical property of this Re-COF hybrid photocatalyst, the TA (ultra-fast transient absorption) and XTA (X-ray transient absorption) including in situ diffuse reflectance UV-Vis-NIR absorption spectrometry techniques were utilized. TA and XTA results indicated that the materials undergo facile intramolecular charge transfer (ICT) through ET (electron transfer) route from photo-excited COF to rhenium moiety, while Re part acted as the CO_2_ reduction catalytic active sites. From the in situ UV-Vis-NIR spectrum results, the authors discovered three key intermediates—TEOA^+^-COF^−^, TEOA^+^-(COF-Re)^−^ and TEOA^+^-COF-Re(CO_2_) adduct—which are responsible for charge separation, induction period and rate-limiting step in the CO_2_ photocatalytic reduction route. Huang’s example was the first COF-homogeneous metal-complex-based hybrid CO_2_ photoreduction catalyst with high activity and selectivity.

**Figure 5:**
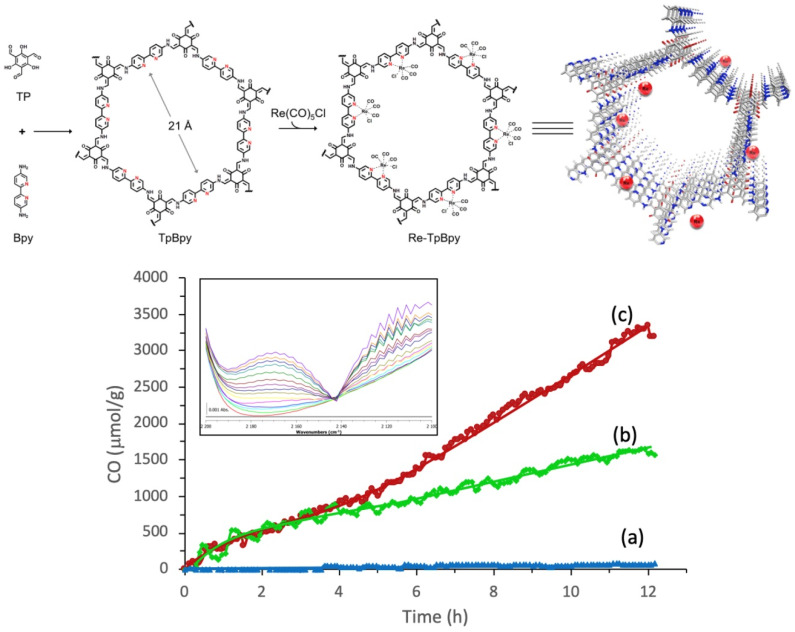
**Top**: Illustration for the construction steps of TpBpy and Re-TpBpy-COF. **Down**: Time course of CO production during photocatalytic CO_2_ reduction on (**a**) TpBpy-COF, (**b**) Re-Bpy, and (**c**) Re-TpBpy-COF photocatalysts under visible light irradiation. Insert: evolution of the CO vibration band during the CO_2_ reduction on Re-TpBpy-COF (time resolution 1 h/spectrum). Copied with permission from Elsevier 2019 [34].

In 2019, Zou et al. studied a highly efficient, stable and recyclable photocatalyst by embedding photoactive rhenium complex (Re(CO)_5_Cl) into bipyridine-based Tp-Bpy COFs [34], as shown in Figure 5. The experimental results of FT-IR, N_2_ isotherm sorption and X-ray diffraction demonstrated that the Re-TpBpy-COF material was constructed as the designed structure. Upon the analysis of the N 1s deconvoluted X-ray photoelectron spectrometry results, it could be confirmed that the pyridinic nitrogen acted as the anchor group for rhenium complex. The as-prepared Re-TpBpy-COF hybrid material showed excellent photocatalytic CO_2_ reduction activity and stability. The CO formation rate was as high as 270.8 μmol g^−1^ h^−1^ for the first cycle under λ ≥ 390 nm irradiation with TEOA as electron donor. Moreover, the Re-TpBpy-COF photocatalyst possessed good recyclability and reusability providing almost negligibly reduced CO evolution activity after 24 h continuous irradiation, while their homogeneous molecular complex photocatalyst counterpart Re(bpy)(CO)_3_Cl showed half the CO formation rate of Re-BpTpy after 4 h irradiation. This example exhibited that the Bpy-based COF material could act as an efficient supporter to anchor photo-active Re-based CO_2_ reduction catalyst. Moreover, the Tp moiety of COF provided extra stabilizing effects due to the keto-enamine tautomerisim-induced conjugation stabilization effect. From the above two examples (Huang and Zou), we can rationalize that Bpy-based COF can be excellent supporter acting similar as non-innocent ligands to render stability for the homogeneous Re-complex molecular photocatalyst, achieving a recyclable and reusable hybrid CO_2_ photocatalytic reduction system. The Re-Bpy-COF based hybrid photocatalyst can be a promising hybrid material system, due to its high activity and stability towards CO_2_ reduction to CO in the presence of an electron donor.

**Figure 6:**
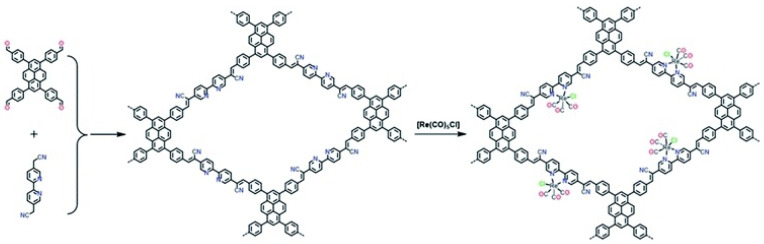
Synthesis of Bpy-sp^2^c-COF and Re-Bpy-sp^2^c-COF. Conditions for Bpy-sp^2^-c-COF: KOH (4 M) 1,2-dichlorobenzene and 1-butanol (1: 1 mixture), 120 °C, 72 h. Copied with permission form Royal Society of Chemistry 2020 [35].

Apart from the above-mentioned BPDA-TTA-COF and Tp-Bpy-COF, Cooper et al., further improved the CO_2_ photocatalytic reduction performance of Re-COF-based materials by introducing pyrene moiety and applying Knoevenagel condensation reaction to fabricate sp^2^-c-COF structure [35], the synthesis process is demonstrated in Figure 6. The as-prepared Bpy-sp^2^-c-COF material could accommodate CO_2_ reduction catalyst Re(CO)_5_Cl by its metal-coordinating Bpy unit. The sp^2^-c conjugation structure led to much better visible-light absorption and charge-separation/transfer ability. Furthermore, the authors utilized steady-state and time-resolved emission spectrometry, time-resolved femto-second transient absorption experimental results to detect the formation of a key long-lived (non-emissive) charge-separation state. Moreover, the DFT calculation results indicated that upon visible-light irradiation, the photo-induced electron dwelled in pyrene fragment would transfer to Bpy fragment and further relay to the complexed Re-based CO_2_ reduction catalyst. This Re-Bpy-sp^2^-c-COF exhibited excellent CO_2_ reduction performance—yielding 1040 μmol g^−1^ h^−1^ and CO over H_2_ selectivity up to 81% using TEOA as sacrificial reagents in acetonitrile solution. This example was, up to now, the record activity of photocatalytic CO_2_ reduction using hybrid COF-homogeneous CO_2_ reduction catalyst hybrid materials.

**Figure 7:**
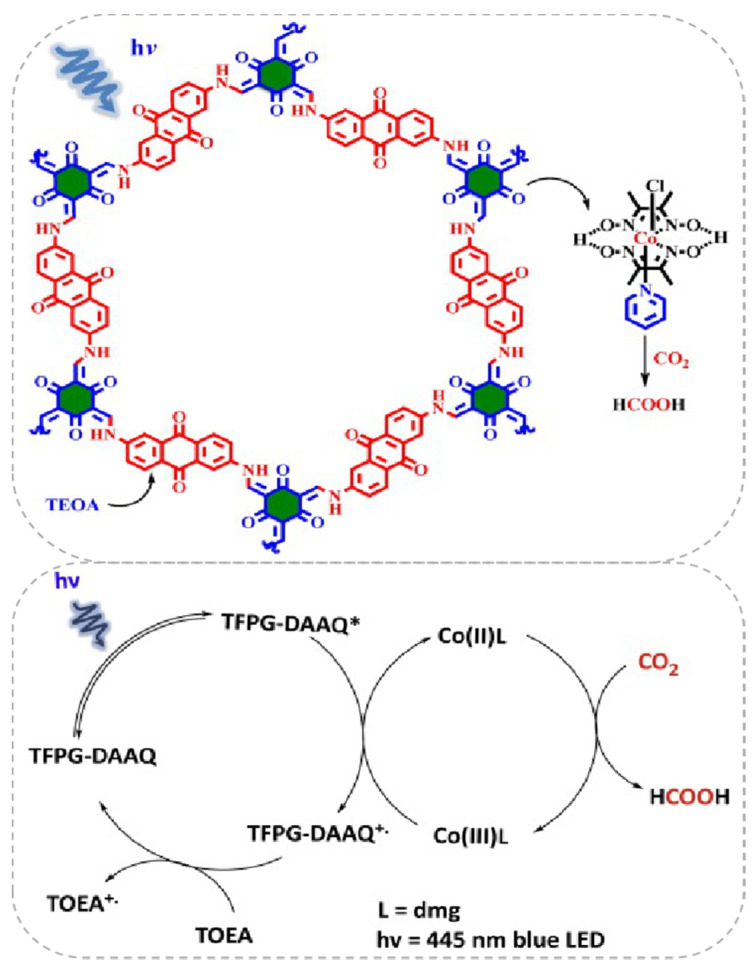
**Top**: Proposed reaction cycle for the photocatalytic CO_2_ reduction using COF and Co(dmg)_2_ as co-catalysts. **Down**: Proposed reaction cycle for the photo-catalytic reduction of CO_2_ into HCOOH. Copied with permission from Elsevier 2019 [36].

Besides the Re-based COF/homogenous molecular CO_2_ photocatalytic reduction system, other earth-abundant transition-metal complexes could also act as efficient components with COF to construct highly effective visible-light induced photocatalytic CO_2_ reduction hybrid material. Very recently, Islam et al. reported that a Co(dmg)_2_ co-catalyst can play pivotal role in assisting TFPG(1,3,5-Triformylphloroglucinol)-DAAQ(2,6-diaminoanthraquinone)-COF for CO_2_ reduction under blue light irradiation (λ = 445 nm) [36], as demonstrated in Figure 7. It was interesting that the CO_2_ photoreduction product was not CO but formic acid. The catalyst system exhibited good activity providing TON up to 125, excellent stability and recyclability without apparent loss of activity for five cycles. The authors proposed a mechanism for this catalytic cycle. Initially, under 455 nm blue light irradiation, only TFPG-DAAQ-COF material was excited because Co(dmg)_2_, TEOA and the product HCOOH absorption spectrum all fall out of this region. After the excitation of COF material to its higher energy state, the electron/hole formation process occurs. The highly-reducing conduction band electron reduces Co(III) to Co(II) complex. The Co(II) complex possessed the ability to coordinate CO_2_ molecules and transformed them into HCOOH by the redox ability of Co(II) metal centre. The oxidized Co(III) species was sequentially reduced by the excited COF molecule. The COF radical cation species was quenched by TEOA to regenerate ground state COF molecule. After irradiation, the newly excited COF molecular re-entered into the next catalytic cycle. This example indicated the possibility to use an earth-abundant CO_2_ reduction co-catalyst to facilitate COF-based photocatalysis.

**Figure 8:**
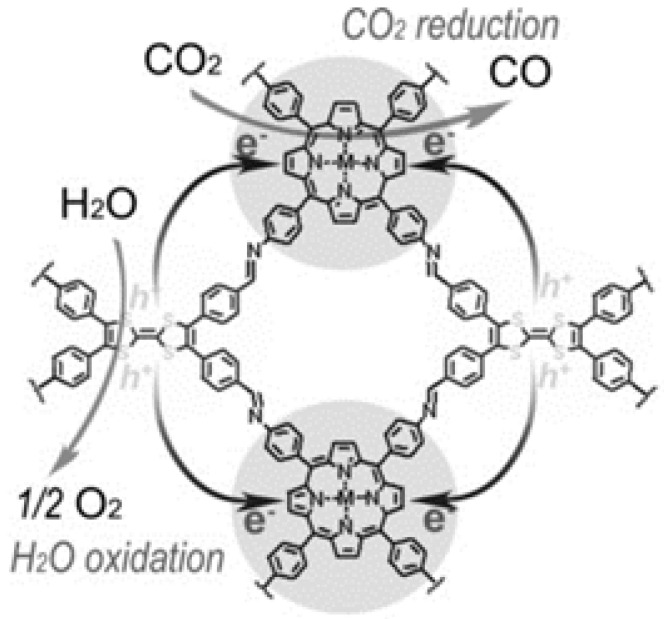
Schematics of the mechanism of TTCOF-M CO_2_RR with H_2_O oxidation. Copied with permission from Wiley 2019 [37].

Lan and co-workers designed and synthesized a series of crystalline porphyrin-tetrathiafulvalene covalent organic frameworks for artificial photosynthesis [37] to combine water oxidation and CO_2_ reduction in a single COF photocatalyst system. The proposed catalysis mechanism is shown in Figure 8. Electron-deficient metalloporphyrin (TAPP) complexes have excellent light harvesting ability and possess potential CO_2_ binding ability, while electron-rich tetrathiafulvalene (TTF) is a superb π-electron donor with rapid electron-transfer property. In this 2D COF material, the strong covalent-coupling effect between TAPP and TTF allows the photo-induced-charges (excitons) to be effectively transferred between TTF and TAPP moieties, resulting that the photo-excited electrons resided on porphyrin and holes on TTF can be effectively exploited for separate reduction and oxidation reaction, respectively. Similar with photosynthesis in natural plant chlorophyll, H_2_O was used as the sole electron-pool for CO_2_ reduction. Especially, for this COF photocatalyst system, there was no need to add extra photosensitizer, sacrificial reagent and noble-metal co-catalyst. By adjusting and optimizing the monomer chemical structure and metal centre of TTCOFs, TTCOF-Zn achieves the highest photocatalytic CO_2_ reduction performance yielding 12.33 μmol CO in 6 h irradiation with about 100% selectivity, while H_2_O acted as final electron-donor and hole-trapping reagent evidenced by the detection of O_2_ evolution. ^13^C and ^18^O isotope-labeling experiments unambiguously indicated that CO was produced from CO_2_ source while O_2_ was from H_2_O. Photoluminescence quenching and time-resolved photoluminescence experiments demonstrated that TTCOF-Zn has more efficient charge-transfer property and longer excited-state life-time for electron-transfer events. From DFT calculation, more direct and clearer evidence for understanding the structure-function relationship of multi-site and multi-electron photocatalytic processes were provided. This discovery is especially significant since only H_2_O and COF are required for photocatalytic CO_2_ reduction without any additive. Moreover, this example provides new perspectives for the design of the next generation crystalline photocatalysts applied for CO_2_ reduction.

### 2.2. Pure COFs CO_2_ Reduction Photocatalysts

In recent years, accompanying the tremendous advance in the field of the synthesis and application of covalent organic frameworks, more and more pure COFs CO_2_ photocatalytic reduction catalysts—without the aid of metal centre or metal-complex CO_2_ reduction co-catalyst, photosensitizer and even sacrificial reagent—have appeared in the research field. The investigation of these pure COFs CO_2_ photocatalytic systems is meaningful, since the application of these pure organic systems represents the future direction for large-scale CO_2_ utilization due to these catalysts being more sustainable and green, without having the secondary environmental effects as the metalated and metal-complex co-catalyzed COFs system do. Although the efficiency of these pure COFs systems is still much lower in comparison with its metalated counterparts, they possess more advantages and long-comings in consideration of sustainability and recyclability. Even though pure COFs CO_2_ photocatalysts are still in their infancy stage, they represent the future direction for COF-based CO_2_ photoreduction catalysts.

**Figure 9:**
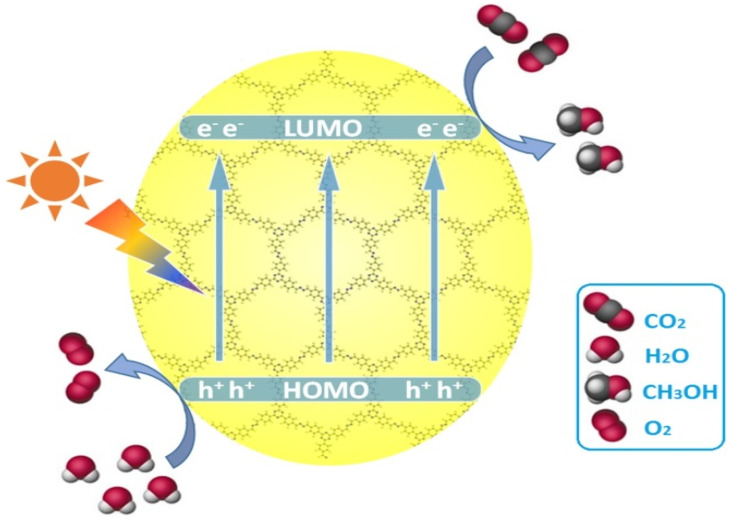
Schematic diagram for the photocatalytic reduction of CO_2_ over azine-based COFs upon visible light irradiation. Copied with permission from Elsevier 2018 [38].

CO_2_ photocatalytic reduction can form various products including CO, HCOOH, methanol, formaldehyde, methane and a small amount of C_2+_ products. By the elaborate design and optimization of COFs monomer and topological alignment, differentiated products other than CO could be yielded in the aqueous photocatalytic CO_2_ reduction by COFs materials. Zhu and co-workers realized metal-free and additive-free photocatalytic CO_2_ reduction by azine-based ACOF and N_3_-COF [38], which is shown in Figure 9. Different from Lan’s TTCOF-Zn, liquid fuel compound methanol was isolated as photo-reduction product by ACOF and N_3_-COF. H_2_O was applied as electron donor although the O_2_ detection experiments were not conducted. The amount of generated CH_3_OH over N_3_-COF catalyst is 13.7 μmol g^−1^ during 24 h irradiation by visible-light, which is more superior to the performance of ACOF and g-C_3_N_4_. The N_3_-COF was revealed to be a robust catalyst possessing high stability in a five-run cycling test of photocatalytic CO_2_ reduction and only exhibited slightly lower yield after first two cycling runs. Moreover, the PXRD peaks, FT-IR and N_2_ adsorption–desorption curves of the used catalyst did not show obvious change after the five-run cycling test compared with the fresh one. Based on the DFT calculation results, N_3_-COF possesses a suitable HOMO and LUMO energy level (−4.95 eV and −2.71 eV) which is both large enough to trigger CO_2_ reduction and small enough to harvest a major portion of visible-light photons. The intramolecular charge transfer event can be induced by photons with wavelengths less than 480 nm. Moreover, the photo-induced hole can be efficiently quenched by H_2_O via hydrogen bond interaction on polar azine moiety, while the photo-induced electron on LUMO can sufficiently reduce CO_2_ on the catalyst surface to methanol due to the matched energy level. Since H_2_O oxidation is a kinetically sluggish process, the COF material is negatively charged. The N_3_-COF demonstrated higher activity due to its electron-withdrawing triazine building block compared with phenyl in ACOF, which can more effectively stabilize the negatively charged intermediate species on COF material. Although the activity of the current example of ACOF and N_3_-COF is not high, the tunability and metal-free property of these COFs-based materials rendered them excellent candidates to supplement current prevalently investigated organic and inorganic semiconductor-based CO_2_ photo-reduction catalysts.

**Figure 10:**
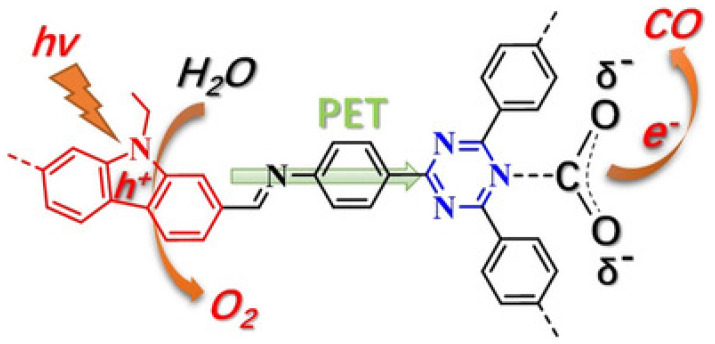
Proposed photoactive electron transfer and reaction pathway in the photoreduction of CO_2_ (PET = photoinduced electron transfer). Copied with permission from Wiley 2019 [39].

Almost at the same time with Lan’s TTCOF photocatalyst system, Kong and co-workers discovered highly effective metal-free donor-acceptor type carbazole-triazine COF photocatalyst, which could photo-reduce CO_2_ to CO using H_2_O as electron source and hole scavenger without any sacrificial reagent or additives [39], as shown in Figure 10. Experimental results showed that CO and O_2_ are the sole products from the visible-light induced photocatalytic reduction of gaseous CO_2_. The generation rate of CO could be optimized to 102.7 μmol g^−1^ h^−1^ with the concomitant formation of stoichiometric amount of O_2_ (51.3 μmol g^−1^ h^−1^). ^13^C and ^18^O isotope tracing experiments exhibited that CO and O_2_ products were actually evolved from the photocatalytic reduction of CO_2_ and oxidation of H_2_O. Moreover, the authors used in situ FT-IR and DFT calculation to expound on the photo-reaction mechanism. This paper possessed important status in COF photocatalytic reduction of CO_2_, since the structure-functionality relationship was well demonstrated in this report. The authors indicated that electron donor-acceptor structure was essential for effective CO_2_ reduction in additive-free COF photocatalysis, which corroborated with Lan’s work using TTCOF to reduce CO_2_.

Very recently, Zhou and co-workers achieved highly efficient photocatalytic reduction of CO_2_ using TAPPB-COF with gaseous water vapor as the sole reductant without any sacrificial reagents and additives in a gas-solid reaction [40]. The authors modified the COF-366 structure by introducing electron-withdrawing bromine group into this porphyrin COF photocatalyst. The introduction of bromine group enhanced the electron density on LUMO according to the DFT calculation results, which contributed to the change of the valence band potential from 0.80 V to 1.10 V (vs. NHE at pH = 7). The up-lifting of the valence band energy greatly facilitated the oxidation of water since the driving force was much larger in comparison with the pristine COF-366 because the redox potential of the pristine COF-366 valence band potential was too close with O_2_/H_2_O (0.82 V vs NHE at pH = 7). The average yield of CO was 24.6 μmol g^−1^ h^−1^ under simulated sunlight irradiation (200 ≤ λ ≤ 1000 nm) for TAPPB-COF, which was about 3 times that of the pristine COF-366 materials and 1.5 times that of the g-C_3_N_4_ yield under other identical conditions. Moreover, not only was the activity of COF photocatalyst enhanced, the selectivity of CO over H_2_ was considerably high (95.6% selectivity). This example indicated that the design and tuning of the electronic property of the monomer unit and material modification via a bottom-up approach could have considerably large influences on the COFs semiconductor valence band (VB) position, which is a key factor for the efficiency of water oxidation semi-reaction. If an appropriate VB potential was generated, the facilitated water oxidation semi-reaction would accelerate the other photo-reduction of CO_2_ semi-reaction.

**Figure 11:**
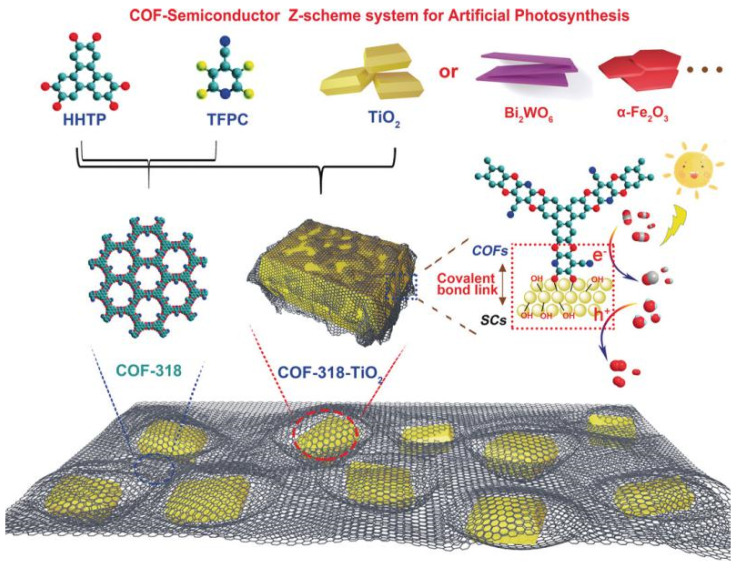
Schematic representation of the preparation of COF-318-SCs via the condensation of COF-318 and semiconductor materials. Copied with permission from Wiley 2020 [41].

Besides pure COF-based CO_2_ photocatalyst, the metal-oxide water oxidation semiconductor could be hybridized to construct Z-scheme heterojunction structure with COF to fulfill efficient overall CO_2_ reduction only with water in the absence of additive, photosensitizer or sacrificial reagent. CO formation yield could be optimized to 69.67 μmol g^−1^ h^−1^ [41], which is the record-high CO_2_ photoreduction performance without photosensitizer, sacrificial reagent and co-catalysts with only water as electron donor. The authors developed COF-316/COF-318 materials by S_N_Ar reaction between aromatic phenol 2,3,6,7,10,11-hexahydroxytriphenylene (HHTP) and two kinds of o-difluoroarenes, i.e., tetrafluorophthalonitrile (TFPN) or 2,3,5,6-tetrafluoro-4-pyridinecarbonitrile (TFPC) as precursor to synthesize two robust PAE-COFs, and the schematic diagram is shown in Figure 11. The as-prepared COF-316/COF-318 was further covalently linked to inorganic semiconductors such as TiO_2_, Bi_2_WO_6_ and α-Fe_2_O_3_ to form COF/inorganic semiconductor hybrid structure. The pyridinecarbonitrile-based COF-318/TiO_2_ provided the best photocatalytic CO_2_ reduction activity creating new record for pure COF-based CO_2_ photocatalytic system. The heterojunction structure was confirmed from transient photocurrent response, photoluminescence and electrochemical impedance spectrometry characterization techniques, these experimental results indicated that COF-318/TiO_2_ catalyst garnered more effective photo-induced charge carrier separation capability and less recombination probability, and much improved electric conductivity in comparison with the semiconductors displaying better charge-transfer property. Moreover, the DFT calculation and in situ XPS experimental results pointed out that upon 365 nm UV irradiation, both COF-318 and TiO_2_ components in COF-318/TiO_2_ were excited. The photo-electrons in TiO_2_ conduction band transferred to COF-318 pyridinic and cyano nitrogen group, which was evidenced from the positive shift binding energy of Ti 2p orbital meaning that the electron density on Ti sites was reduced. Due to the strong adsorption and interaction with CO_2_ molecules at pyridinic and cyano nitrogen sites, the electron was conveniently transferred to CO_2_ coupling with adsorbed proton to yield the key intermediate COOH*. The second proton-coupled-electron-transfer (PCET) was calculated to be the potential-determining-step (PDS). From the DFT results, although the PCET process occurring at cyano nitrogen had a lower over-potential (0.93 eV vs 1.59 eV for pyridinic nitrogen), the first-step activation of CO_2_ to form COF-CO_2_ adduct was more favorable at pyridinic nitrogen sites, which made it possible for the PCET process to occur at both pyridinic and cyano nitrogen sites. The authors demonstrated that the coexistence of pyridinic and cyano group in COF-318 facilitated both CO_2_ activation and reduction processes, which was superior than COF-316 possessing only cyano group. The water oxidation occurred at the TiO_2_ photo-induced VB holes naturally. Lan’s report was the only example to hybrid inorganic semiconductors with COF-based material to fulfill highly effective CO_2_ photoreduction. The delicate Z-scheme type II heterojunction design made it the record-high performance for CO_2_ photoreduction in the absence of photosensitizer, sacrificial reagents and CO_2_ reduction catalyst with only H_2_O as electron donor up to now.

### 2.3. CTF-Organometallic Complex-based CO_2_ Photocatalytic Systems

As relatively similar porous organic polymers compared to covalent organic frameworks, covalent triazine frameworks (CTFs) composed by the triazine units have demonstrated their potential to be utilized as photo-responsive materials and organic semiconductors for a wide range of photochemical applications including water-splitting, CO_2_ reduction and organic transformations due to its nitrogen-rich structure, excellent photo- and chemical stability [42,43].

**Figure 12:**
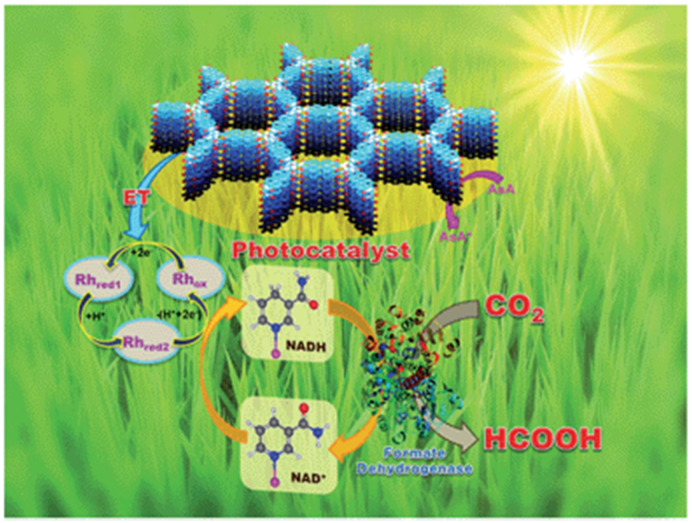
Schematic illustration of the CTF film photocatalyst-enzyme coupled system involved in the exclusive production of formic acid from CO_2_. Rh_ox_ = [Cp * Rh(bpy)H_2_O]^2+^, Rh_red1_ = Cp * Rh(bpy), Rh_red2_ = [Cp * Rh(bpy)H]^+^; Cp* = pentamethylcyclopentadienyl, bpy = 2,2′-bipyridine. Copied with permission from RSC 2016 [44].

As early as 2016, Baeg et al. developed a 2D triazine-based CTF material for photocatalytic CO_2_ reduction application in conjunction with a Rh-complex electrocatalyst, β-NAD^+^ and formate hydrogenase enzyme and ascorbic acid as electron donor [44], as demonstrated in Figure 12. The authors delicately designed and prepared the perylene-based 2D-CTF materials through a co-condensation reaction between cyanuric chloride and perylene diimide, which maneuvered the challenging synthesis of perylene diimide-based carbonitrile monomers. Furthermore, the authors constructed the CTF-Rh-NAD^+^-formate hydrogenase artificial photosynthetic system for CO_2_ reduction with ascorbic acid as sacrificial reagent. The CO_2_ reduction product was HCOOH, with a formation rate as high as 881.3 × 10^3^ μmol gcat^−1^ h^−1^ for the CTF photocatalyst under visible-light irradiation λ ≥ 420 nm at 100 mW cm^−2^ light density. This CO_2_ reduction rate was extremely high. Although the formate hydrogenase enzyme mainly accounted for this extraordinary CO_2_ reduction performance, it could be confirmed that the perylene diimide-based 2D CTF really contributed to the much enhanced visible-light harvesting ability, since the HCOOH formation rate was only 261.85 × 10^3^ μmol gcat^−1^ h^−1^ for the PDI monomer under other identical conditions. According to the cyclic voltammetry experimental results, an electron relay mechanism was proposed. Upon the absorption of visible-light, CTF material was excited. The electron dwelling on HOMO was activated to jump to the LUMO energy level in CTF material. The highly-reducing CTF LUMO electron would transfer to Rh(III) complex. The reduced Rh(I) complex further reduced NAD^+^ to NADPH. The NADPH acted as the reducing reagent for CO_2_ transformation to HCOOH under the formate hydrogenase enzyme catalysis conditions. The oxidized NAD^+^ re-entered into the catalytic cycle and was further reduced by Rh(I) to regenerate Rh(III) and NADPH. NADPH continuously reduced CO_2_ to HCOOH and was recycled by Rh(I) species. The oxidized Rh(III) was reduced by CTF-LUMO electron, while CTF^+^ was quenched by ascorbic acid to regenerate the catalytic active CTF species. This hybrid catalysis assembly unified photocatalysis, transition-metal catalysis, organocatalysis and biocatalysis power to render extremely high CO_2_ reduction efficiency. This example set a benchmark for the further development of CTF- and COF-based photocatalytic CO_2_ reduction applications.

**Figure 13:**
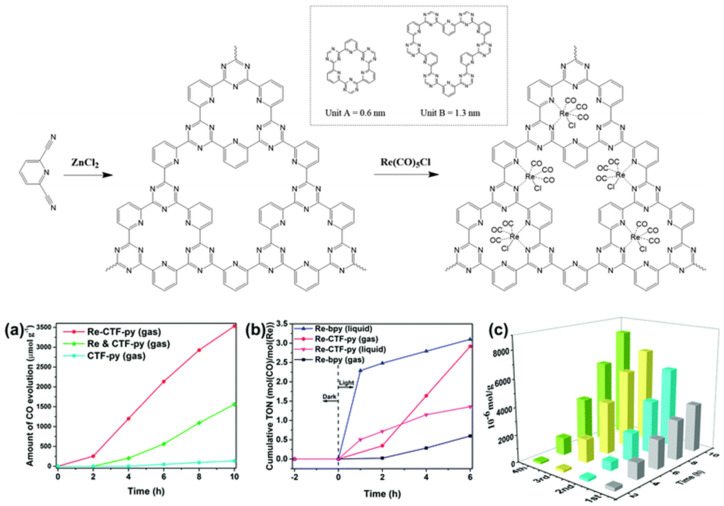
**Top**: Synthesis of CTF-py and Re-CTF-py materials. The inset shows the two types of pores (unit A and unit B). **Down**: (**a**) CO evolution over CTF-py, Re-CTF-py and the physical mixture of CTF-py and Re(CO)_5_Cl (Re and CTF-py) in the solid–gas system. (**b**) Photocatalytic activity of Re-bpy and Re-CTF-py in solid–gas and liquid–gas systems. (**c**) Production yield of CO over the Re-CTF-py photocatalyst to measure its reproducibility by cycling tests. Copied with permission from RSC 2018 [45].

Apart from the CTF-enzyme-based hybrid CO_2_ reduction system, artificial CTF-organometallic composite materials could also act as effective CO_2_ photocatalytic reduction catalysts. Cao et al. developed a pyridine-based CTF material and coordinated a single site Re(CO)_3_Cl CO_2_ reduction catalyst at py-CTF pyridine-triazine bi-dentate chelation sites [45]. The modified Re-py-CTF material showed enhanced CO_2_ photoreduction activity up to a 353.05 μmol g^−1^ h^−1^ formation rate of CO within 10 h under (200 nm–1100 nm) full sunlight spectrum irradiation in a solid–gas system with TEOA vapor as electron donor—see Figure 13. Moreover, the Re-py-CTF-based photocatalyst exhibited much improved photo-stability upon Re leaching. The heterogeneous photocatalyst system provided good recyclability with negligible loss of activity after five consecutive cycles in the solid–gas system. The authors discovered that when soaked in an acetonitrile/TEOA solution, the solid/liquid photocatalytic reaction system showed a much inferior CO formation yield only after the first cycle due to two factors: firstly, the CO_2_ diffusion and mass-transport in the solid-gas system is more efficient than the solid–liquid system because CO_2_ can more effectively transport through the pores of CTF material in solid-gas system while in the solid-liquid system, this mass-transport is hindered partly because the solvent molecules can compete with CO_2_ for the CTF pore adsorption site. Secondly, about half the Re catalyst was leached into the bulk solution in the solid–liquid system. This example demonstrated the important role for CTF-based material in maintaining the activity of homogeneous transition-metal complex CO_2_ reduction catalysts by shielding them from inactivation caused by the leaching effect. This report proved that the solid/gas CO_2_ photoreduction may be preferable over solid/liquid reaction in some cases, especially those involving metal-complex moieties, due to the reduced possibility for catalytic active metal component leaching.

Besides CTF-based materials with noble-metal cocatalysts for photocatalytic CO_2_ reduction, earth-abundant Co could also act as efficient catalytic center to synergistically reduce CO_2_ with CTF under visible-light irradiation. Bi et al. reported that by loading cobalt oxide on CTF-1 material, its performance on CO_2_ reduction to CO was increased by 44-fold in comparison with pristine CTF-1 and 12-fold with Co_3_O_4_ in TEOA/H_2_O/MeCN solution and Ru(bpy)_3_Cl_2_ as photosensitizer [46]. The improvement on catalytic performance is mainly due to the enhanced CO_2_ capture capacity, extended visible-light absorption and efficient transfer of the charge originated from the modification of cobalt.

The performance of the state-of-the-art example of a COF-based photocatalysis system for CO_2_ reduction is summarised in Table 2. To compare the activity of the COF-based photocatalyst for CO_2_ reduction with other semiconductor photocatalyst, we summarized the CO_2_ reduction efficiency by exploiting H_2_O as electron-donor in various systems in Table 3.

## 3. Conclusions

In conclusion, covalent organic frameworks are very promising materials for next-generation photocatalytic CO_2_ reduction applications, since they possess multiple long-comings and advantages over traditional metal-oxide, metal-sulfide, noble-metal plasmonic inorganic semiconductor and inorganic-organic hybrid MOF-based photocatalysts. Firstly, due to COFs materials being composed of organic moieties and non-metal elements, the cost and availability of the COF’s monomer are superior to the metal-containing inorganic semiconductors and MOFs. Secondly, since the electronic and steric factor can be conveniently fine-tuned by the choice of differentiated monomer unit and the reticular chemistry, COFs materials provide us almost unlimited space in which to innovate and modify the structure to be more apt to CO_2_ adsorption and activation, and to enlarge the absorption range of light from UV to Vis and NIR spectrum and to facilitate charge-carrier formation, separation and transfer process to achieve more efficient CO_2_ photoreduction activity and selectivity. Thirdly, due to the property of the linking strong covalent bond, most COFs (except boron-based) are very inert to water, organic solvents, moisture and open-air conditions, with some robust keto-enamine-based Tp-COF being extremely stable and able to endure high-temperature boiling water and concentrated acid and base soaking for long time periods. This property is very different from their metal-containing MOF counterparts, which are woven by weak coordination bonds and most MOFs are unstable in water solution due to that the metal ion are prone to escape from the organic ligand confinement to become free ion in the bulk solution. The stability of COFs in multiple conditions made it promising for the activation of CO_2_ in some extreme conditions. Fourthly, due to its huge surface-area and developed porous structure, a considerably large amount of catalytic active sites and light-harvesting antenna moiety are exposed on the surface in comparison with non-porous inorganic metal-oxide and metal-sulfide photocatalysts. The exposure of active sites promotes the photocatalytic reaction on the surface with the formation of more catalytic active species. Although COFs materials have many such advantages, there are still many gaps to overcome, and challenges to resolve in this research field. Firstly, the extreme difficulty in obtaining a large enough 2D COF single-crystal for common X-ray single crystal diffraction characterization still restricts the further development of 2D COFs multiple applications, including photocatalytic CO_2_ reduction, since the current COF structure elucidation is mainly based on the calculation and the powder X-ray diffraction technique, which relies heavily on experiences, and much uncertainty accompanies the theoretical simulation results. The uncertainty of the COFs structure greatly restricted the elucidation of the SARS in its photocatalytic CO_2_ reaction application. Besides, the CO_2_ reduction rate is still too low. The catalyst activity should be improved from the current μmol g^−1^ h^−1^ scale to mmol g^−1^ h^−1^ and even mol g^−1^ h^−1^. The improvement of catalyst activity calls for deeper investigations into the mechanism of COFs-based CO_2_ photocatalysts. Only if the black-box of the mechanism been deciphered could we design more effective COFs for CO_2_ photoreduction. We are confident that if the above-mentioned issues are soundly addressed, COFs-based materials will have a prosperous and booming future, and become pivotal in settling the CO_2_ reduction problem.

## Figures and Tables

**Table 1:** Reduction potentials of CO_2_.

Reaction	E^0^ (V) vs NHE at pH = 7
2H^+^ + 2e^−^ → H_2_	−0.41
CO_2_ + e^−^ → CO_2_^−^	−1.9
CO_2_ + 2H^+^ + 2e^−^ → HCOOH	−0.61
CO_2_ + 2H^+^ + 2e^−^ → CO + H_2_O	−0.53
CO_2_ + 4H^+^ + 4e^−^ → C + 2H_2_O	−0.2
CO_2_ + 4H^+^ + 4e^−^ → HCHO + H_2_O	−0.48
CO_2_ + 6H^+^ + 6e^−^ → CH_3_OH + H_2_O	−0.38
CO_2_ + 8H^+^ + 8e^−^ → CH_4_ + 2H_2_O	−0.24
2CO_2_ + 8H_2_O + 12e^−^ → C_2_H_4_ + 12OH^−^	−0.34
2CO_2_ + 9H_2_O + 12e^−^ → C_2_H_5_OH + 12OH^−^	−0.33
3CO_2_ + 13H_2_O + 18e^−^ → C_3_H_7_OH + 18OH^−^	−0.32

**Table 2:** The summary of photocatalytic CO_2_ reduction performance for COFs with different structure.

Type	COFs	Light	Additive	Conditions	Product Yield	Selectivity (%)	Ref
Enamine	DQTP-COF-Co	λ ≥ 420 nm 300 W Xe lamp	Ru(bpy)_3_Cl_2_/TEOA	Liquid (MeCN)	CO: 1.02 × 10^3^ μmol·g^−1^·h^−1^	59.4	[31]
Imine	COF-367 -Co NS	λ ≥ 420 nm 300 W Xe lamp	Ru(bpy)_3_Cl_2_/ascorbic acid	Liquid (MeCN)	CO: 10,672 μmol·g^−1^·h^−1^	78	[32]
Enamine	TpBp-COF-Ni	λ ≥ 420 nm 300 W Xe lamp	Ru(bpy)_3_Cl_2_/TEOA	Liquid (H_2_O)	CO: 966 μmol g^−1^ h^−1^	96	[33]
Imine	Re-COF	λ ≥ 390 nm 300W Xe lamp	TEOA	Liquid (MeCN)	CO:780 μmol·g^−1^·h^−1^	98	[34]
Enamine	Re-TpBpy COFs	λ ≥ 390 nm 200 W Xe lamp	TEOA	Liquid (MeCN/H_2_O)	CO: 270.8 μmol·g^−1^ h^−1^	N/A	[35]
Olefin	Bpy-sp2-c-COF	λ ≥ 420 nm 300 W Xe lamp	TEOA	Liquid (MeCN)	CO: 1040 μmol·g^−1^ h^−1^	81	[36]
Enamine	TFPG-DAAQ-COF	λ = 445 nm blue LED	TEOA	Liquid (MeCN)	HCOOH: TOF = 6	N/A	[37]
Imine	TTCOF-Zn	420–800 nm 300 W Xe lamp	None	Liquid (H_2_O)	CO: 2.055 μmol·g^−1^ h^−1^	100	[38]
Azine	N_3_-COF	420–800nm 500 W Xe lamp	None	Liquid (H_2_O)	CH_3_OH:0.57μmol·g^−1^ h^−1^	N/A	[39]
Imine	TT-COF	λ > 420 nm 300 W Xe lamp	None	Solid-gaseous H_2_O	CO: 102.7 μmol·g^−1^·h^−1^	98	[40]
Imine	TAPPB-COF	200–1000 nm Xe lamp	None	Solid-gaseous H_2_O	CO: 24.6 μmol·g^−1^·h^−1^	95.6	[41]
Arylether	COF-318-SCs	380–800 nm 300 W Xe lamp	None	Solid-gaseous H_2_O	CO: 69.67 μmol·g^−1^·h^−1^	N/A	[42]
Triazine	CTF	λ ≥ 420 nm 450 W Xe lamp	β-NAD^+^/Rh complex/formate hydrogenase/ ascorbic acid	Liquid (Na_3_PO_4_ aq. solution)	HCOOH: 881.3 × 10^3^ μmol·g^−1^·h^−1^	N/A	[45]
Triazine	Re-CTF-py	200–1100 nm 300 W Xe lamp	TEOA	Solid–gas	CO: 353.05 μmol·g^−1^·h^−1^	N/A	[46]

**Table 3:** The summary of photocatalytic CO_2_ reduction efficiency using H_2_O as electron-donor for COFs and other semiconductor materials [47,48,49,50,51,52,53,54,55,56,57,58,59,60,61].

Photocatalyst	Light Source	Product Yield μmol·g^−1^ h^−1^	Ref
TTCOF-Zn	420–800 nm 300 W Xe lamp	CO: 2.055	[38]
N_3_-COF	420–800 nm 500 W Xe lamp	CH_3_OH: 0.57	[39]
TT-COF	Λ > 420 nm 300 W Xe lamp	CO: 102.7	[40]
TAPPB-COF	200–1000 nm Xe lamp	CO: 24.6	[41]
COF-318-SCs	380–800 nm 300 W Xe lamp	CO: 69.67	[42]
CPO-27-Mg/TiO_2_	UV lamp 4 W 365 nm	CO: 4.09, CH_4_: 2.35	[47]
Well-crystallized ordered mesoporous TiO_2_	UV-Vis 300W Xe lamp	CO: 0.145, CH_4_: 0.195	[48]
TiO_2_	300 W Xe lamp (λ ≥ 408 nm)	CO: 50.7, CH_4_: 13.5	[49]
Co-ZIF-9/TiO_2_	UV-Vis 300 W Xe lamp (200 < λ < 900)	CO: 17.58	[50]
BiOBr	300 W Xe lamp (λ ≥ 400 nm)	CO: 87.4	[51]
Defect-Rich Bi_12_O_17_Cl_2_ Nanotubes	300 W Xe lamp	CO: 48.6	[52]
ZIF-8/C_3_N_4_	300 W full-spectrum Xe lamp	CH_3_OH: 0.75	[53]
QS-Co_3O4_ (ZIF-67)	200 W Xe lamp (AM 1.5)	CO: 46.3	[54]
Bi_4_O_5_I_2_/g-C_3_N_4_	300 W Xe lamp (λ ≥ 400 nm)	CO: 45.6, CH_4_: 6	[55]
Z-scheme CdS– WO_3_	300 W Xe lamp (λ > 420 nm)	CH_4_:1.02	[56]
HCP-TiO_2_-FG	300 W Xe lamp (λ ≥ 420 nm)	CO: 27.62, CH_4_: 21.63	[57]
α-Fe_2_O_3_/g-C_3_N_4_	300 W Xe lamp (λ≥ 420 nm)	CO: 27.2	[58]
C-TiO_2_-x@g-C_3_N_4_	300 W Xe lamp (λ ≥ 420 nm)	CO: 205	[59]
TiO_2_/N-doped-RGO	400 W Xe lamp (λ = 250–400 nm)	CO: 44.56	[60]
TiO_2_/NH_2_-UiO-66	1500 W Xe lamp (λ > 325 nm	CO: 4.25	[61]

## References

[1] McConnell I., Li G., Brudvig G.W. (2010). Energy Conversion in Natural and Artificial Photosynthesis. Chem. Biol..

[2] Heindel N.D., Pfau M.A. (1965). A profitable partnership: Giacomo Ciamician and Paul Silber. J. Chem. Educ..

[3] Albini A., Fagnoni M. (2008). 1908: Giacomo Ciamician and the concept of green chemistry. ChemSusChem.

[4] Chang X., Wang T., Gong J. (2016). CO_2_ photo-reduction: Insights into CO_2_ activation and reaction on surfaces of photocatalysts. Energy Environ. Sci..

[5] Kim W., Edri E., Frei H. (2016). Hierarchical Inorganic Assemblies for Artificial Photosynthesis. Acc. Chem. Res..

[6] Li K., Peng B., Peng T. (2016). Recent Advances in Heterogeneous Photocatalytic CO_2_ Conversion to Solar Fuels. ACS Catal..

[7] Li Z., Feng J., Yan S., Zou Z. (2015). Solar fuel production: Strategies and new opportunities with nanostructures. Nano Today.

[8] Inoue T., Fujishima A., Konishi S., Honda K. (1979). Photoelectrocatalytic reduction of carbon dioxide in aqueous suspensions of semiconductor powders. Nature.

[9] Wang F., Li Q., Xu D. (2017). Recent Progress in Semiconductor-Based Nanocomposite Photocatalysts for Solar-to-Chemical Energy Conversion. Adv. Energy Mater..

[10] Jiao W., Shen W., Rahman Z.U., Wang D. (2016). Recent progress in red semiconductor photocatalysts for solar energy conversion and utilization. Nanotechnol. Rev..

[11] Lingampalli S.R., Ayyub M.M., Rao C.N.R. (2017). Recent Progress in the Photocatalytic Reduction of Carbon Dioxide. ACS Omega.

[12] Marszewski M., Cao S., Yu J., Jaroniec M. (2015). Semiconductor-based photocatalytic CO_2_ conversion. Mater. Horiz..

[13] Ola O., Maroto-Valer M.M. (2015). Review of material design and reactor engineering on TiO_2_ photocatalysis for CO_2_ reduction. J. Photochem. Photobio. C-Photochem. Rev..

[14] Sinopoli A., La Porte N.T., Martinez J.F., Wasielewski M.R., Sohail M. (2018). Manganese carbonyl complexes for CO_2_ reduction. Coord. Chem. Rev..

[15] Yamazaki Y., Takeda H., Ishitani O. (2015). Photocatalytic reduction of CO_2_ using metal complexes. J. Photochem. Photobio. C-Photochem. Rev..

[16] Sun Z., Talreja N., Tao H., Texter J., Muhler M., Strunk J., Chen J. (2018). Catalysis of Carbon Dioxide Photoreduction on Nanosheets: Fundamentals and Challenges. Angew. Chem. Int. Ed..

[17] Yang J. (2018). Progress of Metal Oxide (Sulfide)-Based Photocatalytic Materials for Reducing Nitrogen to Ammonia. J. Chem..

[18] Li R., Zhang W., Zhou K. (2018). Metal-Organic-Framework-Based Catalysts for Photoreduction of CO_2_. Adv. Mater..

[19] Dhakshinamoorthy A., Asiri A.M., Garcia H. (2016). Metal-Organic Framework (MOF) Compounds: Photocatalysts for Redox Reactions and Solar Fuel Production. Angew. Chem. Int. Ed..

[20] Chen Y., Wang D., Deng X., Li Z. (2017). Metal-organic frameworks (MOFs) for photocatalytic CO_2_ reduction. Catal. Sci. Technol..

[21] Cote A.P., Benin A.I., Ockwig N.W., O’Keeffe M., Matzger A.J., Yaghi O.M. (2005). Porous, crystalline, covalent organic frameworks. Science.

[22] Diercks C.S., Yaghi O.M. (2017). The atom, the molecule, and the covalent organic framework. Science.

[23] Diercks C.S., Lin S., Komienko N., Kapustin E.A., Nichols E.M., Zhu C., Zhao Y., Chang C.J., Yaghi O.M. (2018). Reticular Electronic Tuning of Porphyrin Active Sites in Covalent Organic Frameworks for Electrocatalytic Carbon Dioxide Reduction. J. Am. Chem. Soc..

[24] Ding X., Guo J., Feng X., Honsho Y., Guo J., Seki S., Maitarad P., Saeki A., Nagase S., Jiang D. (2011). Synthesis of Metallophthalocyanine Covalent Organic Frameworks That Exhibit High Carrier Mobility and Photoconductivity. Angew. Chem. Int. Ed..

[25] Feng X., Liu L., Honsho Y., Saeki A., Seki S., Irle S., Dong Y., Nagai A., Jiang D. (2012). High-Rate Charge-Carrier Transport in Porphyrin Covalent Organic Frameworks: Switching from Hole to Electron to Ambipolar Conduction. Angew. Chem. Int. Ed..

[26] Diercks C.S., Liu Y., Cordova K.E., Yaghi O.M. (2018). The role of reticular chemistry in the design of CO_2_ reduction catalysts. Nat. Mater..

[27] Guo L., Jin S. (2019). Stable Covalent Organic Frameworks for Photochemical Applications. Chemphotochem.

[28] Banerjee T., Gottschling K., Savasci G., Ochsenfeld C., Lotsch B.V. (2018). H-2 Evolution with Covalent Organic Framework Photocatalysts. ACS Energy Lett..

[29] Ma D., Wang Y., Liu A., Li S., Lu C., Chen C. (2018). Covalent Organic Frameworks: Promising Materials as Heterogeneous Catalysts for C-C Bond Formations. Catalysts.

[30] Lu M., Li Q., Liu J., Zhang F.-M., Zhang L., Wang J.-L., Kang Z.-H., Lan Y.-Q. (2019). Installing earth-abundant metal active centers to covalent organic frameworks for efficient heterogeneous photocatalytic CO_2_ reduction. Appl. Catal. B-Environ..

[31] Liu W., Li X., Wang C., Pan H., Liu W., Wang K., Zeng Q., Wang R., Jiang J. (2019). A Scalable General Synthetic Approach toward Ultrathin Imine-Linked Two-Dimensional Covalent Organic Framework Nanosheets for Photocatalytic CO_2_ Reduction. J. Am. Chem. Soc..

[32] Zhong W., Sa R., Li L., He Y., Li L., Bi J., Zhuang Z., Yu Y., Zou Z. (2019). A Covalent Organic Framework Bearing Single Ni Sites as a Synergistic Photocatalyst for Selective Photoreduction of CO_2_ to CO. J. Am. Chem. Soc..

[33] Yang S., Hu W., Zhang X., He P., Pattengale B., Liu C., Cendejas M., Hermans I., Zhang X., Zhang J. (2018). 2D Covalent Organic Frameworks as Intrinsic Photocatalysts for Visible Light-Driven CO_2_ Reduction. J. Am. Chem. Soc..

[34] Li S.-Y., Meng S., Zou X., El-Roz M., Telegeev I., Thili O., Liu T.X., Zhu G. (2019). Rhenium-functionalized covalent organic framework photocatalyst for efficient CO_2_ reduction under visible light. Micropor. Mesopor. Mater..

[35] Fu Z., Wang X., Gardner A., Wang X., Chong S.Y., Neri G., Cowan A.J., Liu L., Li X., Vogel A. (2020). A stable covalent organic framework for photocatalytic carbon dioxide reduction. Chem. Sci..

[36] Sarkar P., Riyajuddin S., Das A., Chowdhury A.H., Ghosh K., Islam S.M. (2020). Mesoporous covalent organic framework: An active photo-catalyst for formic acid synthesis through carbon dioxide reduction under visible light. Mol. Catal..

[37] Lu M., Liu J., Li Q., Zhang M., Liu M., Wang J.-L., Yuan D.-Q., Lan Y.-Q. (2019). Rational Design of Crystalline Covalent Organic Frameworks for Efficient CO_2_ Photoreduction with H_2_O. Angew. Chem. Int. Ed..

[38] Fu Y., Zhu X., Huang L., Zhang X., Zhang F., Zhu W. (2018). Azine-based covalent organic frameworks as metal-free visible light photocatalysts for CO_2_ reduction with H_2_O. Appl. Catal. B Environ..

[39] Lei K., Wang D., Ye L., Kou M., Deng Y., Ma Z., Wang L., Kong Y. A Metal-Free Donor–Acceptor Covalent Organic Framework Photocatalyst for Visible-Light-Driven Reduction of CO_2_ with H_2_O. ChemSusChem.

[40] Wang L., Wang R.-L., Zhang X., Mu J.-L., Zhou Z.-Y., Su Z.-M. Tuning the VB of COF to improve the efficiency of photoreduction of CO_2_ with water. ChemSusChem.

[41] Zhang M., Lu M., Lang Z.-L., Liu J., Liu M., Chang J.-N., Li L.-Y., Shang L.-J., Wang M., Li S.-L. (2020). Semiconductor/Covalent-Organic-Framework Z-Scheme Heterojunctions for Artificial Photosynthesis. Angew. Chem. Int. Ed..

[42] Liu M., Guo L., Jin S., Tan B. (2019). Covalent triazine frameworks: Synthesis and applications. J. Mater. Chem. A.

[43] Zhang Y., Jin S. (2019). Recent Advancements in the Synthesis of Covalent Triazine Frameworks for Energy and Environmental Applications. Polymers.

[44] Yadav R.K., Kumar A., Park N.-J., Kong K.-J., Baeg J.-O. (2016). A highly efficient covalent organic framework film photocatalyst for selective solar fuel production from CO_2_. J. Mater. Chem. A.

[45] Xu R., Wang X.-S., Zhao H., Lin H., Huang Y.-B., Cao R. (2018). Rhenium-modified porous covalent triazine framework for highly efficient photocatalytic carbon dioxide reduction in a solid-gas system. Catal. Sci. Technol..

[46] Bi J., Xu B., Sun L., Huang H., Fang S., Li L., Wu L. (2019). A Cobalt-Modified Covalent Triazine-Based Framework as an Efficient Cocatalyst for Visible-Light-Driven Photocatalytic CO_2_ Reduction. ChemPlusChem.

[47] Wang M., Wang D., Li Z. (2016). Self-assembly of CPO-27-Mg/TiO_2_ nanocomposite with enhanced performance for photocatalytic CO_2_ reduction. Appl. Catal. B Environ..

[48] Wang T., Meng X., Li P., Ouyang S., Chang K., Liu G., Mei Z., Ye J. (2014). Photoreduction of CO_2_ over the well-crystallized ordered mesoporous TiO_2_ with the confined space effect. Nano Energy.

[49] Yaghoubi H., Li Z., Chen Y., Ngo H.T., Bhethanabotla V.R., Joseph B., Ma S., Schlaf R., Takshi A. (2015). Toward a Visible Light-Driven Photocatalyst: The Effect of Midgap-States-Induced Energy Gap of Undoped TiO_2_ Nanoparticles. ACS Catal..

[50] Yan S., Ouyang S., Xu H., Zhao M., Zhang X., Ye J. (2016). Co-ZIF-9/TiO_2_ nanostructure for superior CO_2_ photoreduction activity. J. Mater. Chem. A.

[51] Wu J., Li X., Shi W., Ling P., Sun Y., Jiao X., Gao S., Liang L., Xu J., Yan W. (2018). Efficient Visible-Light-Driven CO_2_ Reduction Mediated by Defect-Engineered BiOBr Atomic Layers. Angew. Chem. Int. Ed..

[52] Di J., Zhu C., Ji M., Duan M., Long R., Yan C., Gu K., Xiong J., She Y., Xia J. (2018). Defect-Rich Bi_12_O_17_Cl_2_ Nanotubes Self-Accelerating Charge Separation for Boosting Photocatalytic CO_2_ Reduction. Angew. Chem. Int. Ed..

[53] Liu S., Chen F., Li S., Peng X., Xiong Y. (2017). Enhanced photocatalytic conversion of greenhouse gas CO_2_ into solar fuels over g-C_3_N_4_ nanotubes with decorated transparent ZIF-8 nanoclusters. Appl. Catal. B Environ..

[54] Wang L., Wan J., Zhao Y., Yang N., Wang D. (2019). Hollow Multi-Shelled Structures of Co_3_O_4_ Dodecahedron with Unique Crystal Orientation for Enhanced Photocatalytic CO_2_ Reduction. J. Am. Chem. Soc..

[55] Bai Y., Ye L., Wang L., Shi X., Wang P., Bai W., Wong P.K. (2016). g-C_3_N_4_/Bi_4_O_5_I_2_ heterojunction with I_3_^−^/I^−^ redox mediator for enhanced photocatalytic CO_2_ conversion. Appl. Catal. B Environ..

[56] Jin J., Yu J., Guo D., Cui C., Ho W. (2015). A Hierarchical Z-Scheme CdS–WO_3_ Photocatalyst with Enhanced CO_2_ Reduction Activity. Small.

[57] Wang S., Xu M., Peng T., Zhang C., Li T., Hussain I., Wang J., Tan B. (2019). Porous hypercrosslinked polymer-TiO_2_-graphene composite photocatalysts for visible-light-driven CO_2_ conversion. Nat. Commun..

[58] Jiang Z., Wan W., Li H., Yuan S., Zhao H., Wong P.K. (2018). A Hierarchical Z-Scheme α-Fe_2_O_3_/g-C_3_N_4_ Hybrid for Enhanced Photocatalytic CO_2_ Reduction. Adv. Mater..

[59] Zhou J., Wu H., Sun C.-Y., Hu C.-Y., Wang X.-L., Kang Z.-H., Su Z.-M. (2018). Ultrasmall C-TiO_2−x_ nanoparticle/g-C_3_N_4_ composite for CO_2_ photoreduction with high efficiency and selectivity. J. Mater Chem. A.

[60] Lin L.-Y., Nie Y., Kavadiya S., Soundappan T., Biswas P. (2017). N-doped reduced graphene oxide promoted nano TiO_2_ as a bifunctional adsorbent/photocatalyst for CO_2_ photoreduction: Effect of N species. Chem. Eng. J..

[61] Crake A., Christoforidis K.C., Kafizas A., Zafeiratos S., Petit C. (2017). CO_2_ capture and photocatalytic reduction using bifunctional TiO_2_/MOF nanocomposites under UV–vis irradiation. Appl. Catal. B Environ..

